# Comparative evaluation of various analgesics in reducing pain in irreversible pulpitis

**DOI:** 10.6026/97320630017313

**Published:** 2021-02-28

**Authors:** Pradeep Solete, Sindhu Ramesh

**Affiliations:** 1 Department of Conservative Dentistry and Endodontics, Saveetha Dental College, Saveetha University, Saveetha Institute of Medical and Technical Sciences, Chennai 600077, India

**Keywords:** Analgesics, Inter appointment pain, Placebo, Root canal therapy, Symptomatic irreversible pulpitis

## Abstract

It is of interest to evaluate a single dose of three different analgesics compared to placebo in patients with symptomatic irreversible pulpitis. 120 patients were enrolled with severe pain in this prospective clinical trial. Patients were randomly divided into four groups after shaping and cleaning of root canals. This includes placebo, piroxicam 20mg, acetaminophen 325mg with aceclofenac sodium 100mg and acetaminophen 650mg. Participants were given a questionnaire to note the pain scores at various time intervals (6 hrs, 12 hrs, and 24 hrs) along with the respective tablets in a concealed manner. Data thus collected was analyzed for statistical significance. The severity of pain decreased in all the three interventional groups compared to the control group (p <0.01) at 6 hours. Zerodol-P and dolonex showed better pain reduction in comparison to the placebo and dolo 650 group (p <0.05) at 12 and 24 hours. Data shows that both zerodol-P and dolonex groups had similar effects at all time intervals. Thus, a single dose of analgesic such as Zerodol-P and Dolonex following shaping and cleaning of root canals relieved pain at all time intervals of the treatment. However, Dolo 650 performed better during the initial 6hrs after completion of the shaping and cleaning of root canals compared to the placebo.

## Background

Pulpitis can be defined as the inflammation of the pulp mainly due to cariogenic reasons and less often due to trauma and restorative treatment. Kim et al (1990) described this process of inflammation of the pulp [1]. The major reasons for the pain are due to the release of inflammatory mediators, which activates the nociceptors surrounding the tooth [2]. Ng et al showed that the patient experiences pain after completing the endodontic treatment is about 3-58%. Postoperative pain following endodontic therapy is due to acute inflammation of the periapical tissues due to instrumentation, irrigants and debris extrusion. The inflammatory mediators released following noxious stimulus are prostaglandins, especially PGE2, which can cause hyperalgesia, vasodilatation, and increase vascular permeability [3]. The main action of Non-steroidal anti-inflammatory drugs (NSAID) consists of decreasing the inflammation thereby down- regulating the activity of cyclo-oxygenase (COX) enzymes which exist in two iso-forms namely COX-1 and COX -2. Traditional NSAIDs acts by non-selective inhibition of COX activity thereby resulting in some gastrointestinal side effects [4]. Aceclofenac sodium is a phenylacetic acid derivative and a potent analgesic and anti-inflammatory agent. It inhibits the action of COX enzyme, which is involved in the synthesis of prostaglandins [5]. The combination of NSAID and acetaminophen showed an additive effect in controlling dental pain. Piroxicam is an NSAID that is commonly used to relieve post-operative dental pain. The main action is by inhibiting the COX enzymes and also suppresses thromboxane synthesis in platelets, which hinders the secondary phase of platelet aggregation [6]. Patients often have post-operative pain up to 24 hours after root canal treatment [7]. Pain is subjective in nature and the threshold differs in each individual. In this study the assessment of pain intensity was performed using the Visual analog scale (VAS) of (0-10) [8]. Thus, the aim of this study is to evaluate various analgesics in irreversible pulpitis to eliminate pain.

## Materials and Methods:

One hundred and twenty patients enrolled for this prospective clinical trial, which was endorsed by the institutional ethical committee (IHEC/SDMDS11ODS7). The sample size was determined based on a pilot study of 40 cases (10 per group), using G Power 3.1.2 version, and details as follows: power of 0.9 and p<0.05, the sample size arrived were 27 per group, for the compensation of drop outs during follow-up, sample size was set at 30 per group. The criteria for the enrollment as follows: Patients aged from 18-45 years with no medical problems, no history of medication used 12 hrs prior to presenting for the treatment, patients with irreversible pulpitis with no signs of apical periodontitis, patients with severe pain (7-10) in 10 points visual analog scale. The pulpal status of the affected tooth was subjected to sensibility testing (Thermal test and Electric Pulp tester). Patient with one tooth of irreversible pulpitis with severe pain was selected for the clinical trial. Informed consent was taken from all the participants. A trained person carried out randomization before the beginning of the trial, using a table of random numbers with block sizes being unknown to the investigators. SNOSE (sequentially numbered, opaque, sealed envelopes) method was employed for the allocation concealment. A piece of paper containing a randomized number was sealed in the opaque cover containing the serial number, which was done by a person who was not associated in this trial. The sealed envelope was opened once the intervention was assigned. The enrolled participants were divided randomly into 4 groups respectively; Group 1 - Placebo (gelatin capsule) Group, Group 2 - Dolonex ( Piroxicam 20mg; Pfizer Limited, India), Group 3- Zerodol-P (Aceclofenac sodium 100mg with Acetaminophen 325mg;IPCA Laboratories Limited, Mumbai, India), Group 4 - Dolo 650 (Acetaminophen 650mg; Micro Labs Limited, Bangalore, India). The preoperative and postoperative pain scores were recorded using 10 points visual analog scale (VAS), No pain (Score - 0), Mild pain (Scores 1-3), Moderate Pain (Scores 4-7), Severe pain (Scores 7-10).

## Protocol for the Treatment:

The principal investigator following standard protocol performed treatment in all cases. Patient consent was obtained and the tooth was anesthetized using 2% lignocaine with 1:200000 epinephrine solution, followed by standard access cavity was prepared under rubber dam isolation and the occlusal reduction was done. The length of all the canals was measured by an electronic apex locator (Root ZX, J Morita corp., Kyoto, Japan) and verified using radiographs. Root canal preparation was done in a crown down manner using Ni-Ti rotary instruments (ProTaper universal system, Dentsply Sirona, Swiss). All the canals were instrumented till size 25 of Ni-Ti rotary instruments 0.5mm short of working length. Copious irrigation was done using 3% Sodium Hypochlorite solution (Prime dental, India) and 17% Ethylenediaminetetraacetic acid (EDTA) (RC help, Prime dental, India) to facilitate the instrumentation. Canals were dried using paper points and the access cavity was sealed with temporary restorative material using Cavit (3M ESPE, St Paul, MN, USA). A single dose of interventional drug was given for the respective patients, along with a rescue medication of Ketorolac 10mg (Dr. Reddys Laboratories LTD, India), was given at the end of first visit and the patient was instructed to call up the evaluator before taking the tablet.

## Assessment of pain intensity following treatment:

A questionnaire containing a pain scale (VAS) was given after the treatment to record the intensity of pain at 6, 12 and 24 hours respectively. At the end of this trial endodontic treatment was completed.

## Statistical Analysis:

Normal Distribution data assessment was done by the Kolmogorov- Smirnov test. The obtained values were analyzed statistically using one-way ANOVA comparing baseline and the other time periods. Independent t-test was done to assess the performance of all drugs at all time intervals (statistically significant when p<0.05) using SPSS 20 (SPSS inc., Chicago, IL, USA).

## Results:

**Figure 1:**
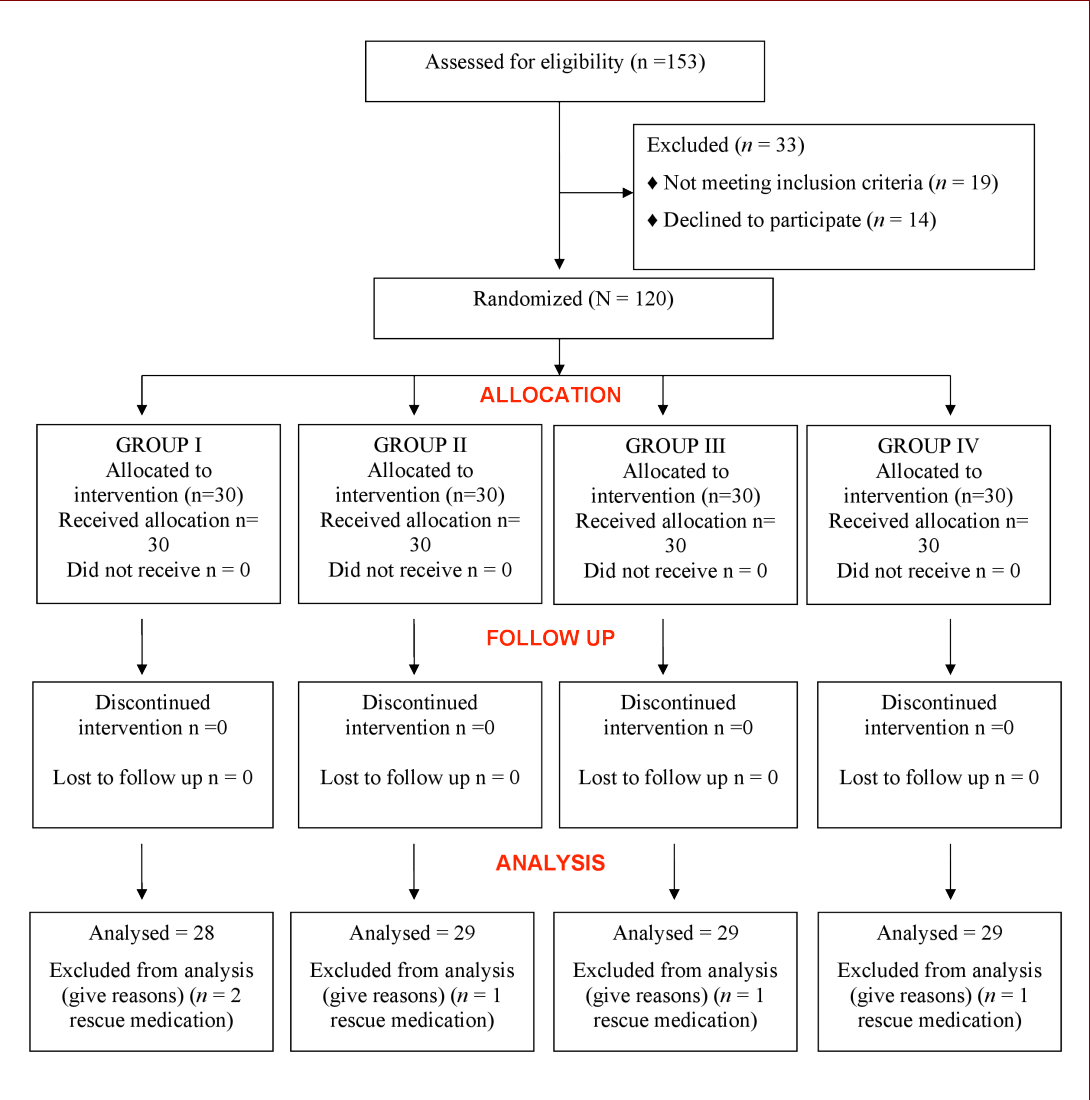
Consort 2010 flowchart for the trial

**Figure 2:**
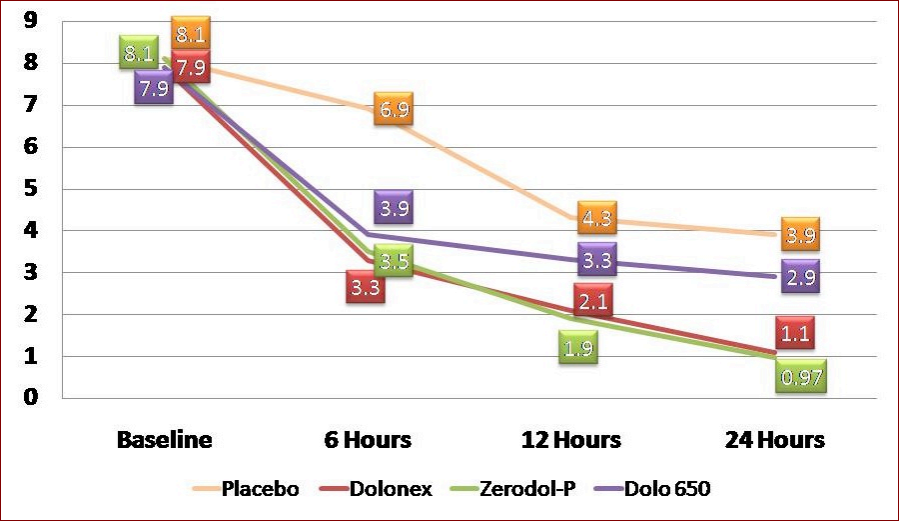
Pain reduction at all time intervals.

Over a period of 19 months, 120 participants were selected upon receiving the informed consent for the study. The demographic data were reported. Totally 5 patients did not take into account: 2 from Placebo, 1 each from the other three groups. The reason was they had taken a rescue medication as they experienced severe pain (Figure 1). The Analysis was carried out based on duly filled forms at all intervals using VAS for 115 participants (Table 1). Baseline scores of all patients showed no significant difference among all groups. One-way Anova showed there is some significant difference after trial commencement. Independent t-test was done to assess the performance of all drugs at all time intervals. At 6 hours interval, all the interventional groups resulted in significant pain reduction when compared to the placebo group (p<0.01). At 12 hours and 24 hours interval group II and group III showed a significant pain reduction compared to the other two groups (p<0.05), with no significant pain reduction either of the two at all time intervals. No statistical significant difference was seen amongst group I and group IV at 12 and 24 hours (p>0.05) (Figure 2: Pain Reduction at all time intervals). The percentage of pain reduction calculated using Pre-treatment VAS score – Postoperative VAS score) X 100 / Pre-treatment VAS score at each time interval (6 hours, 12 hours and 24 hours). In this study, Dolonex (group II) and Zerodol-P (Group III) showed higher pain reduction compared to the other two groups (Table 2).

## Discussion:

In this prospective controlled trial we chose three different analgesics to assess the pain reduction following shaping and cleaning of root canals in patients with symptomatic irreversible pulpitis. Only patients presenting with severe pain (VAS >7-10) were enrolled. Controlling this pain is often a difficult task for the clinician. The endodontic treatment was performed in a crown down manner, the advantages are- less extrusion of debris thereby having less postoperative pain, reduction of microorganisms pushing towards apical areas, easier smear layer removal with the help of chelating agents, enhanced disinfection of the entire canals thereby facilitating the irrigant flow [9]. ProTaper universal Nickel-titanium instruments were used (Dentsply Sirona, Swiss). ProTaper universal rotary system consists of shaping files and finishing files. SX is designed to flare root canal orifice, S1 and S2 were used to shape the coronal third and middle third of the root canal. Finishing file F1 and F2 were used to clean the apical third and to enlarge the middle third of the canals [10]. Standardized Irrigation protocol was followed: 3% Sodium hypochlorite, 17% EDTA and normal saline were used. After shaping and cleaning of the canals the access cavities were sealed with temporary restorative material [11]. The postoperative pain was less in root canals instrumented with ProTaper universal when compared to wave one reciprocating file [12]. In this study we used placebo, the inclusion of these has some significant clinical relevance, as many clinical studies showed post operative pain has reduced without any interventional drugs [9], our study showed 47% of pain reduction at the end of 24 hrs. A single dose of interventional drug was given to the respective patients, as most of the patients experienced the pain within 24 hrs of the treatment procedures [7]. The result of this study showed that all the interventional groups showed significant pain reduction compared to placebo groups (p<0.05) at 6 hours interval. After 12 hours and 24 hours significant pain reduction was seen in group II (Dolonex) and group III (Zerodol-P) when compared to group IV (Dolo 650) and group I (Placebo) (Table 2). Totally 5 patients were not taken into account from this study as they had taken rescue medications within 12 hours (2 from Placebo, 1 each from the other three groups). The NSAIDs impedes the release of inflammatory mediators, thereby reducing the pain, especially moderate to severe pain after endodontic treatment [13]. Acetaminophen is a de-ethylated active metabolite of phenacetin introduced in 1950. It inhibits prostaglandin synthesis in the CNS by interacting with serotonin and nitric oxide mechanisms [14]. The plasma half-life found to be 2-3 hours, the patients enrolled in this group had pain reduction effectively at 6 hours interval compared to the control group (p<0.05). At 12 and 24 hours, there was no significant difference when compared to the control group (p>0.05). The pain reduction (%) for this group was 58% and 63 % at the end of 12 and 24 hours respectively. Aceclofenac is a phenylacetic acid compound derived from a chemical designation of [2-{(2, 6-dichlorophenyl) amino}-phenylacetoxyacetic acid). It is plasma half-life is 4 to 5 hours. The mechanism involved in pain reduction are as follows: a) decreases the inflammation activity, b) down-regulates the inflammatory mediators IL-1b and TNF, c) decreases the activity of basal and IL-1b-stimulated IL-6 production, d) inhibits cyclo-oxygenase activity, e) inhibits PGE2 production, f) reduces the stimulated generation of reactive oxygen species, and g) interferes with expression of cell adhesion molecules [15]. In a study done by Kundaravalli et al. [16], Aceclofenac showed better pain relief in comparison to diclofenac in the treatment of postoperative extraction dental pain. A systematic review done by Vohra et al. [17], Aceclofenac showed better pain relief in musculoskeletal pain when compared to Diclofenac. In our study, the pain reduction reported after consuming Aceclofenac at 12 hours and 24 hours was 73.4% and 88% respectively. Piroxicam (Dolonex 20mg) is an oxicam class of drug, a non-selective COX inhibitor, with a meanshelf life of 50-60 hours. This drug possesses analgesic and antipyretic action thereby permits once-daily dosing. In the present study pain reduction reported after consumption of Piroxicam at 12 hours and 24 hours was 76% and 88% respectively. Premedication with piroxicam showed less orthodontic separator pain when compared to ibuprofen and placebo [18]. Post endodontic pain was assessed by Joshi et al 2017 using piroxicam either orally or Intra-ligamentary compared to placebo. However piroxicam showed better pain relief, of which intra-ligamentary was performed, the reason could be oral piroxicam has to undergo hepatic bypass before reaching the target site [19]. The placebo group in our study showed a 50% pain reduction at the end of 24 hours. NSAIDs are extremely useful in reducing post-operative pain in dentistry. The use of piroxicam (Dolonex) and Zerodol-P showed promising results in reducing the post-operative pain of 86% and 88% respectively at the end of 24 hours.

## Conclusion

We used three different analgesics in comparison to placebo. The results showed that a single dose of analgesic such as Zerodol-P and Dolonex for shaping and cleaning of root canals relieved pain at all time intervals of the treatment. However, Dolo 650 performed better during the initial 6 hrs after completion of the shaping and cleaning of root canals compared to the placebo.

## Figures and Tables

**Table 1:** Pain scores at all time interval

	Group I	Group II	Group III	Group IV	P value
Baseline	8.1 ± 0.6	7.9 ± 0.8	8.1 ± 0.9	7.9 ± 0.6	P>0.05
6 Hours	6.9 ± 1.6	3.3 ± 0.8	3.5 ± 0.9	3.9 ± 1.2	P<0.01
12 Hours	4.3 ± 1.2	2.1 ± 0.6	1.9 ± 0.8	3.3 ± 0.9	P<0.05
24 Hours	3.9 ± 0.8	1.1 ± 0.4	0.97 ± 0.5	2.9 ± 1.2	P<0.05

**Table 2:** Pain reduction at all time intervals

Time intervals	Group I - Placebo	Group II - Dolonex	Group III - Zerodol-P	Group IV - Dolo 650	p value
Pain reduction at 6 hours	13.50%	58.20%	56.70%	50.60%	p<0.01
Pain reduction at 12 hours	46.91%	73.40%	76%	58%	p<0.05
Pain reduction at 24 hours	47.19%	86%	88%	63%	p<0.05
